# Waterscapes domestication: an alternative approach for interactions among humans, animals, and aquatic environments in Amazonia across time

**DOI:** 10.1093/af/vfab019

**Published:** 2021-06-19

**Authors:** Gabriela Prestes-Carneiro, Roberta Sá Leitão Barboza, Myrian Sá Leitão Barboza, Claide de Paula Moraes, Philippe Béarez

**Affiliations:** 1 Anthropology and Archaeology Program, Institute of Social Sciences, Federal University of Western Para (UFOPA), Santarém, Pará,Brazil; 2 Laboratory of Teaching, Research and Fisheries Extension for Amazonian Communities (LABPEXCA), Institute of Coastal Studies (IECOS), Federal University of Pará (UFPA), Bragança, Pará,Brazil; 3 Archéozoologie, archéobotanique: sociétés, pratiques et environnements (AASPE, UMR 7209), CNRS, Muséum national d′histoire naturelle, Paris,France

**Keywords:** Amazonia, animal domestication, archaeology, water technologies, waterscape

**Implications**

- In this article, we propose the “waterscape domestication” concept as a way to understand how humans and animals have interacted throughout history in the many aquatic environments in Amazonia.
- To support our proposal, we present and discuss historical and contemporary cases of interactions between forest people and animals in waterscapes. We describe archaeological structures and management practices of ponds, dams, and turtle and fish corrals.
- Through archaeological, ethnohistorical, ethnoecological and ethnographic studies we show the domestication concept should be broadened to include the worldview of forest people and their interactions with Amazonian waterscapes.

## Introduction 

Animal management and domestication have been widely interpreted and studied in relation to terrestrial mammals; however, there are still debates over what “domestication” means for aquatic animals. Fishing has deep archaeological roots, as early as the Paleolithic (e.g., Cleyet-Merle, 1990; Braun et al., 2010; Fujita et al., 2016). There is solid evidence that specialized fish production and management began around 3500 BC in freshwater systems in China (Malindine, 2019), but there is less solid evidence that it may have begun in the same country as early as 6000 BC (Nakajima et al., 2019). Egyptian tomb art suggests management of the Nile tilapia (*Oreochromis niloticus*) around 1500 BC (Harache, 2002). In Europe, the common carp (*Cyprinus carpio*) was farmed by the Romans about 2,000 years ago but was not domesticated until medieval times (Balon, 2004).

Forest people have interacted with and managed aquatic and terrestrial environments across time in Amazonia, yet, compared with the Middle East and European centers of faunal domestication, few animals in Lowland South America, specifically Amazonia, have been domesticated. The term “forest people,” as many of the traditional people in Latin America recognize themselves, is not used here in the literal sense since it does not only refer to people who inhabit forested environments, but it also refers to multiple collectives and human groups that commonly held lands and natural resources; Allegretti, 1989; Almeida, 2008. In this way, this term encompasses traditional people from rainforest, dry forest, and palm forest, liana forest, savanna, wetland, and several others. In the Andes, the classic examples of domestication are the llama (*Lama glama*), alpaca (*Vicugna pacos*), and guinea pig (*Cavia porcellus*) (Stahl, 2008). As defined by the naturalist Wallace (1858), the traditional concept of domestication is related to human supremacy in the control of nonhuman species (Barboza, 2019). For biologists, the domestication process generally implies modification of a species’ genetic heritage. This develops a novel set of morphological features known as the “domestication syndrome” (Harlan, 1992). The only case of classical animal domestication that could have taken place in the tropical lowlands of South America during the same period is that of the Muscovy duck (*Cairina moschata*) (Stahl, 2005).

Although classical animal domestication (Stépanoff and Vigne, 2018) was lacking in the Amazon, many wild species have been kept as pets (in pet-keeping relationships, wild animals are captured and adopted but they are neither breed nor consumed by local people; Erikson, 2012), and recent studies suggest that human groups significantly altered Amazonian landscapes. A possible factor of animal non-domestication in the lowlands is how indigenous Amazonians interact with animals (Stahl, 2014). Inspired by Viveiros de Castro (1998) and Descola (1986, 2013), Stahl (2014) postulates that indigenous people eschewed subjugating animals to a position of dependence and subordination. Several of altered landscapes may be related to structures designed to keep aquatic animals in captivity or to “enhance the natural habitats of wild fish to increase their availability” (Erickson, 2008, p. 174). This seems to be the case of pre-Columbian and modern structures (i.e., ponds, corrals, dams, artificial wetlands, raised field canals, causeways, and other water management techniques) associated with fish and water management (Erickson, 2000, 2008; Lombardo and Prümers, 2010; Blatrix et al., 2018).

Studies have highlighted the importance of the domestication process in Amazonia, but most of them are restricted to populations of terrestrial plants and animals (Viveiros de Castro, 1998). An exception is Sautchuk (2007), who calls attention to relationships among humans and aquatic animals, mainly fish. Therefore, we aim to broaden domestication concepts based on the evidence of physical structures that demonstrate interactions between humans and waterscapes in Amazonia.

It should be recognized that the relationship between indigenous people and aquatic environments, albeit not specifically in relation to domestication, was earlier documented in the literature (Steward, 1948; Sauer, 1952); however, the idea of waterscape domestication is nonetheless under-emphasized. To support our proposal, we first provide a brief conceptual background for the concept of waterscape domestication, and, second, we provide a repertoire of historical and recent cases of interaction of forest people with animals in waterscapes, describing archaeological structures and management practices. Finally, we discuss how aquatic environments and their constituents—animals and water—have been modified through interaction with humans. We also consider that humans can be modified in these interactions, but it is not our intention to enter into a more detailed discussion of this aspect in the present text.

## Amazonian Hydrology

Amazonia is the region that is drained by the Amazon River and its tributaries together with adjacent lowlands (Balée, 2003). The Amazon region is not uniform but is composed of different environments, such as floodplains, upland forests, savannas or (*llanos*), and mangroves, each of which has its particularities (Moran, 1990). Floodplains (also known as *várzeas*) are low-relief areas near large rivers, which are periodically flooded. Floodings are due to the lateral overflow of rivers or lakes and by the rain or groundwater-flood pulse (Junk et al., 1989). Generally, there is a 6-month rainy season where plains are flooded, however they can remain inundated for the majority of the year. Floodplains are environments of high biological productivity due to the large amount of suspended material carried by the Amazon River and the presence of floating aquatic plants (Sioli, 1984). Upland forests (or *terra firme*) are the highest areas of the Amazon and are not flooded. The Amazonian savannas—known as cerrado in Brazil, and *Llanos* in Colombia and Venezuela—are mainly situated in the boundaries of the Amazon region. They have a hyper-seasonal regime, with a strong dry season and a strong wet season, which creates extensive areas of flooded savanna. The llanos generally have poor drainage of the soil, which causes water to stagnate for months (Moran, 1990). Mangroves are marginal and unique ecosystems, defined by daily tide variations and sea (salt water) influence (Vannucci, 2001). Each one of these environments has a different relation with water, even in non-inundated landscapes such as the upland forests has aquatic landscapes such as streams. All the environments change every year, with the arrival of the rainy season. The volume of water, the speed at which it arrives, and the elements that it brings (animals, plants, nutrients, and salinity) shape and modify the landscapes. The observation of these dynamics is crucial to understand human-made water structures and, therefore, the structures described below need to be understood based on the environmental context in which they are embedded.

## Conceptual Background for Waterscape Domestication

Waterscape concepts gained theoretical shape and became widely used from the work of Swyngedouw (1999). As an aspect of political ecology, Swyngedouw (1999) uses the term “waterscapes” to emphasize the hybrid character of the aquatic landscape and to highlight that nature and society are deeply intertwined. Swyngedouw (1999) investigates the water politics and engineering in Spain’s modernization process and shows that Spanish waterscapes and societies embody a multiplicity of historical–geographical relations and process. According to him, social interactions and power relations coproduce waterscapes.

Inspired by Swyngedouw’s conceptualization of waterscapes, various and complementary conceptual framings have been used. Some of them encompass local cosmologies, knowledge, and identity, as well as the connection between the land, water, humans, and nonhuman beings. Strang (2005), for instance, argues the importance of sensory, aesthetic, and imaginative dynamics in people–water interactions, fundamental for the constitution of social identity. She demonstrated that the cultural meaning of water—or “fluidscapes’’ as she prefers to call— among Aboriginal societies in Australia—is intimately related to identity construction. 

Here, we adopt the concept of waterscapes based on its meaning and value, and in recognition of forest people ontology and encounters among multiple beings, as stressed by Gagné and Rasmussen (2016, p. 138):

In short, the concept of waterscape, as it has been developed at the crossroads between political ecology and studies of science, is useful for grasping how places are produced in uneven encounters and how water distribution and equity (or lack thereof) are fundamental features of these encounters. Furthermore, the concept leads us to further nuance these questions by examining a variety of ways of knowing and interacting with water in different waterscapes.

Additionally, Gagné and Rasmussen (2016) highlight the boundaries and interfaces between water and land based on “amphibious anthropology” framework. Societies involved in these landscapes are nurtured and disrupted by the changing flow of water (Gagné and Rasmussen, 2016). It is important to recognize the confluence of land and water and the influence of the flow of water in these landscapes. Short-term fluctuations and seasonal variations provoked by water movements—either by rainy season or by the influence of the moon—are crucial elements in waterscape dynamics in which humans are engaged. 

Previous literature on domestication, especially anthropological and historical ecology studies (Teletchea and Fontaine, 2012; Tsing, 2012), has criticized the traditional discourse on domestication that assumed the notion of human control over a passive nature (Smith, 1995). Besides this, researchers complain about the intrinsic idea of a defined frontier between “wild” and “domesticated” species (Levi-Strauss, 1952). Based on these epistemological problems in the domestication discourse, scholars working in Amazonia recommend replacing the domestication concept with alternative notions, such as antidomestication, familiarizing predation, co-domestication, mutual-domestication, and several others (Morim de Lima, 2017; Fausto and Neves, 2018; Carneiro da Cunha, 2019). However, none of them debate the cases of human interaction with aquatic animals and environments.

In relation to “domestication of water,” archaeologists Mithen (2010) and Garfinkel et al. (2006) refer to this concept by arguing that the development of water management in Late Neolithic populations fostered the emergence of ancient cities in Jordan Valley. They found archaeological remains of cisterns, wells, dams, aqueducts, a system of extensive series of structures for plant irrigation, and various water supply management structures in several parts of Jordan. This notion considers humans as the main manipulator and transforming agent of the natural properties of water for his own needs (Mithen, 2010).

Although the original meaning of domestication may have a connotation of “domination,” Macauley (2005) addresses that domestication has another interpretation and contributes to better understanding of our relations with technology and the aquatic environment. According to Macauley (2005, p. 168), “water also carries and conducts values to us” and “domestication is also cognate with *domus* (house or home), thus rendering something very particular—in this case water—known and relatively familiar on an everyday basis.”

In the present paper, we prefer to readapt the “domesticated landscape” concept and we propose the term “waterscape domestication” to describe the interactions among humans, other beings (here, we refer to beings of multiple natures and morphologies—plants, stones, and spirits; Viveiros de Castro, 1998), and waterscapes in the Amazon and to extend the notion of “domesticated landscape” to aquatic environments. The “domesticated landscape” was first defined by Yen (1989) and Clement (1999,pp. 191–192), and Erickson (2008, p. 158) reviewed the concept. According to Erickson (2008, p. 158):

Domestication of landscape implies all intentional and non-intentional practices and activities of humans that transform the environment into a productive landscape for humans and other species. Domesticated landscapes are the result of careful resource creation and management with implications for the diversity, distribution, and availability of species. Through their long-term historical transformation of the environment involving transplanting of plants and animals, selective culling of non-economic species and encouragement of useful species, burning, settlement, farming, agroforestry (forest management), and other activities discussed in this paper, humans created what we recognize and appreciate as nature in Amazonia. Through the perspective of historical ecology, however, we see that nature in Amazonia more closely resembles a garden than a pristine, natural wilderness. Rather than “adapt to” or be “limited by” the Amazonian environment, humans created, transformed, and managed cultural or anthropogenic (human-made) landscapes that suited their purposes. The cultural or anthropogenic landscapes range from the subtle (often confused with “natural” or “pristine”) to completely engineered.

Clement and Cassino (2018) indicates that Amazonia, as well as all continents with human societies, has a mosaic of landscapes with different degrees of domestication. A sequence of categories of landscapes was classified by Clement and Cassino (2018) according to the intensity of landscape intervention and manipulation. A detailed classification of the degrees of intervention in waterscapes, in the parameters proposed by Clement (1999) for landscape, is an important work to come. However, for now, we suggest that different interventions can be perceived in terms of the longevity and durability of their brands in the environment. 

In addition to terrestrial management, Erickson (2008) also included water management (river cutoffs, transportation and communication networks, and water control) and fisheries management as elements of a domesticated landscape. Considering this proposition and drawing from reflections about waterscapes interactions and complexity, we examine how waterscapes and their constituents have been managed as a complex and integrated system. The notion of waterscapes should not always be associated with the idea that water is abundant, as there are many relatively dry interfluvial areas in Amazonia. What we emphasize here is that indigenous peoples managed the waterscapes using the water when it was available. The concept also considers the longitudinal dimension of rivers, with water movements (flooding, broadening of streams, and receding waters events) and fish migrations.

## Archaeological and Historical Evidence of Managed Structures

**Figure 1.**
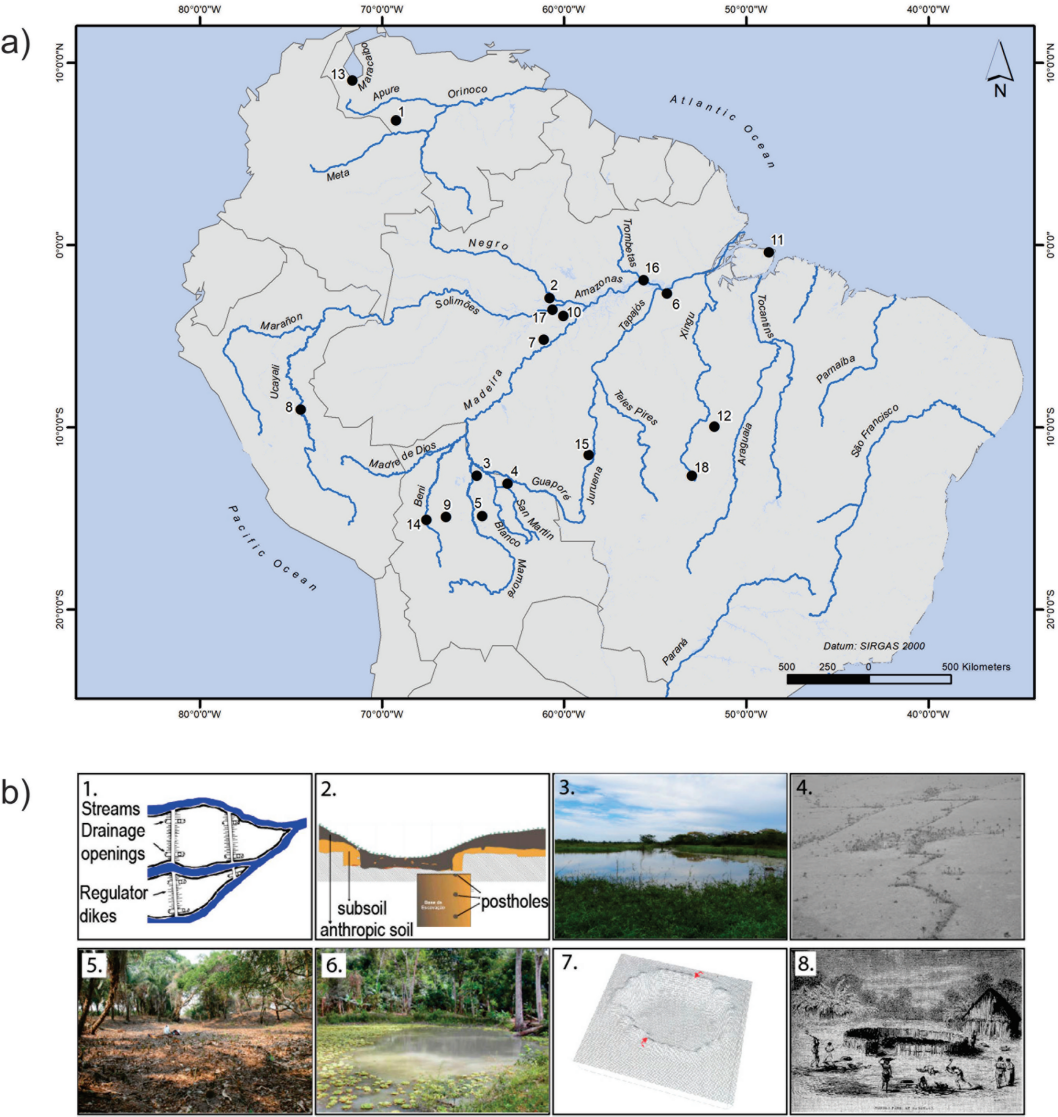
(a) Map showing the distribution of water management systems in the archaeological and historical record. The ecosystem in which the structure is placed follows in parenthesis. (b) Images of the structures (1) Water retention systems from the Apere savannas in the Venezuelan Llanos: ~AD 500 to AD 1400 (Zucchi, 1984) (savannas). (2) Archaeologically observed artificial ponds in Lago do Limão site, in the central Brazilian Amazon interpreted as turtle corrals: ~AD 300 to AD 1200 (Moraes, 2006) (floodplain). (3) Archaeological ponds associated with raised fields in the Exaltacion área, Bolivia: ~AD 400 to AD 1400 (Rodrigues et al., 2017) (savanna). (4) Archaeological earthen fish weirs associated with ponds in Baures, Bolivia: ~AD 1000 to AD 1300 (Erickson, 2000; Blatrix et al., 2018) (savanna). (5) Archaeological ponds nearby earthen platform sites (Lomas) in Trinidad, Bolivia: ~AD 1000 to AD 1200 (Lombardo and Prümers, 2010; Prestes-Carneiro et al., 2019) (savanna). (6) Thirty-five archaeological ponds recorded on the Belterra Plateau, Brazil: ~AD 1300 to AD 1400 (Nimuendajú, 1952; Stenborg et al., 2018; Troufflard and Travassos, 2019) (uplands). (7) An archaeological pond at the Guajará site near Borba, Brazil: ~AD 1000 (Moraes, 2013) (floodplain). (8) Turtle corrals that were used by the Conibo in the Ucayali, Peru: 19th century (Marcoy, 1875) (unknown). (9) Archaeological pond associated with raised field in the San Borja área, Bolivia; no data available (Iriarte and Dickau, 2012) (savanna). (10) River dams observed by Nimuendajú (2004) between AD 1922 and AD 1924 reported in Rio Preto do Pantaleão in the region of Autazes, Brazil (unknown). (11) Seasonally flooded, dug depressions associated with archaeological occupations of raised platforms, or *Tesos*, on the Marajó Island, Brazil: ~AD 500 to AD 700 (Schaan, 2008; Schaan et al., 2010) (mangrove). (12) Artificial obstructions of river courses and ponds, possibly related to fishing, in the área of the Upper Xingú River, Brazil: ~AD 1200 to present (Heckenberger et al., 2003) (unknown). (13) Modern construction of stone dams and weirs in rivers by the Bari groups that occupy the southwesternmost lobe of the Maracaibo, Venezuela (Beckerman, 1983) (unknown). (14) Modern construction of stone and clay dams by Tacana groups in Llanos de Mojos, Bolivia (Hissink and Hahn, 2000) (savanna). (15) Modern wooden fish weirs constructed seasonally by the Enanewe-Nawe in tributaries of the Juruena River, Brazil (Mendes dos Santos and Santos, 2008) (uplands-savanna transition). (16) Ponds dug at springs and in stream channels at the Cipoal do Aaticum archaeological site, Trombetas River, Brazil: ~AD 900 to AD 1400 (Schmidt et al., 2014) (uplands). (17) Canals and ponds recorded at the Laguinho archaeological site, in the Central Amazon, Brazil: ~ AD 600 to AD 1100 (Schmidt et al., 2014) (floodplain). (18) Dams, reservoirs, and ponds observed while conducting fieldwork in the Upper Xingu that await mapping and dating (Schmidt et al., 2014) (savanna).

Unlike most of the domesticated mammals, such as pigs or cattle, fish raised in ponds do not appear to have undergone morphological changes in their anatomy. Thus, the evidence of “confinement” is noticeable mainly in archaeological landscapes and ethnohistorical and ethnographic accounts (Figure 1). In the Amazon Basin, there is a dearth of archaeological research concerning what structures are connected to fishing and water storage and what their function may have been. This contrasts with the information available on the Brazilian Atlantic coast (Nery, 1995), the Amazon River estuary (Bezerra, 2017), Atlantic coast of southeastern and southern Brazil (Noelli et al., 1995; Borges, 2016), Pacific Coast (Favier Dubois et al., 2019), and the Andes (Lane, 2014).

### Artificial ponds

The first mentions of artificial ponds in the Amazon appear in the reports of the first Europeans to navigate along the Amazon River. Friar Gaspar de Carvajal mentions the abundance of fish, turtles, manatees, and birds found in the villages they passed. At one point, he mentions “[...] there was great food, there were turtles in corrals and water huts, meat, and fish and *bizcocho*, and they were in such abundance that a total of a thousand men could eat for a year [...] “(Carvajal, [1542] 1942, p. 27). However, from an archaeological and ethnographic point of view, these structures are still little known. In numerical terms, the Bolivian Amazon region is, until now, the place with the largest number of artificial ponds. In the Baures region, between the Guaporé and Mamoré rivers, more than 382 ponds have been recorded (Blatrix et al., 2018). Southeast of the Baures region, Prümers (2007) identified a set of artificial ponds near monumental platforms (*lomas*) in the Llanos de Mojos region, Bolivia. The ponds found at the Loma Salvatierra archaeological site are approximately 30 m wide and 2 m deep (See Figure 1(5)). A core sample from one of the ponds was taken and a layer of clay loam, rich in organic matter, was interpreted to be the bottom of the pond and delivered calibrated radiocarbon dates between AD 1000 and AD 1200 (Lombardo and Prümers, 2010).

**Figure 2.**
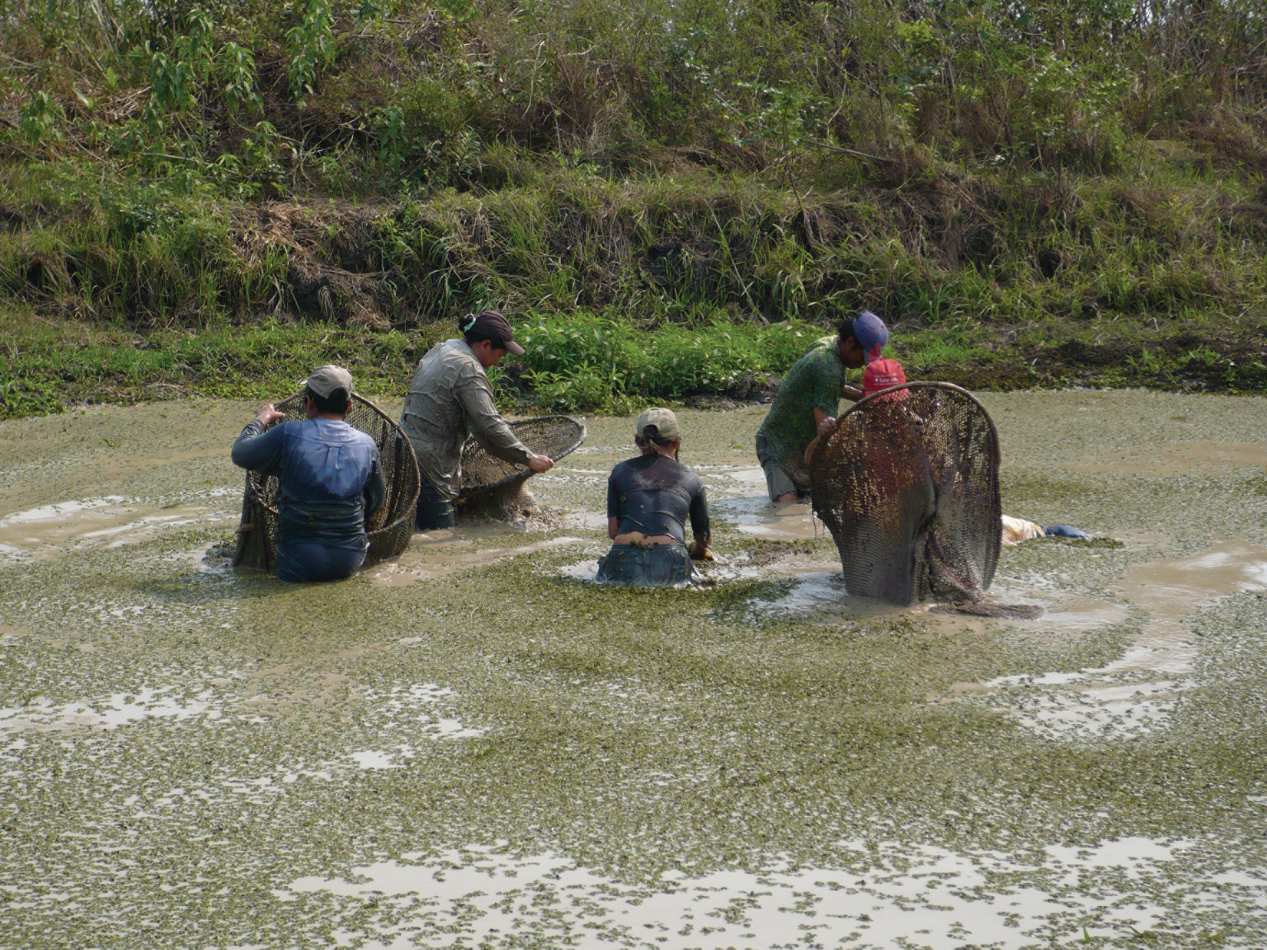
Fishing with cotton nets in an artificial pond. Baures, Bolivia (Credits: Franciska Reidel).

The archaeological fish fauna from the Loma Salvatierra site is composed mainly of small-sized fishes, including undetermined small sardines (Characidae), *pirañas* (Serrasalmidae) and *serepapas* (Cichlidae), swamp-eels (*Synbranchus* spp.), and lungfishes (*Lepidosiren paradoxa*). These species are quite resistant to aquatic environments with low oxygen conditions and are often found in modern artificial ponds in the region. These facts suggest that the function of these structures was to store water and fish (Prestes-Carneiro et al., 2019). In the Llanos de Mojos region, there are ponds associated with raised fields in the Exaltación region (Iriarte and Dickau, 2012; Rodrigues et al., 2017); however, they have been neither dated nor studied (see Figure 1(3)). Nowadays, such ponds, both ancient and modern, are exploited by local women who use cotton fishing nets to capture available aquatic species (see Figure 2).

In the Central Amazon, there are several natural lakes that are seasonally affected by the alternating flood dynamics in the basins of the Negro and Amazon rivers. Several archaeological sites are strategically located near these lakes. In Lago do Limão (Moraes, 2006, 2013), this dynamic can radically transform the landscape, sometimes supplying the lake with black waters from the Negro river (most of the year) or with muddy waters from the Amazon river. In addition, controlling the periods of extreme flow in the lake system is a guarantee of abundant fishing. Digging or reestablishing channels to access parts of the system that are becoming disconnected is a task still performed by the populations living in the region. In the lake areas, it is still common that for a few days at the end of the low-water season, fishermen from traditional communities gather in large numbers for the “days of the fish with their heads out” (Moraes, 2006).

This current dynamic makes it difficult to identify which of these structures may be related to the same period of the archaeological sites, since structures managed today may only be the continuity of management. In any case, Schmidt et al. (2014) suggest that some excavated ponds documented in the floodplains near the Laguinho site (Central Amazon) may be associated with the occupation of the site.

Artificial ponds are found in the Belterra Plateau (~2,000 km^2^). Of the 68 recorded archaeological sites, 35 have artificial or natural ponds principally located near streams, yet far from large rivers (Stenborg, 2016). However, the only pond systematically excavated by researchers was located near the Cedro site (see Figure 1(6)). The pond is approximately 1.1 m deep and 12 m wide. On the banks of this pond, clay balls were found that seemed to have served as support for the walls of the pond (Troufflard and Travassos, 2019). The Cedro site, like most of the sites on the Belterra Plateau, is dated between AD 1300 and AD 1400 (Nimuendajú, 1952; Stenborg et al., 2018; Troufflard and Travassos, 2019). In the ponds of Belterra, no faunal remains were preserved and there was no zooarchaeological investigation.

In the lower Madeira river, at the Guajará archaeological site, Moraes (2013) mapped an oval hollow, 38 × 32 m in size, 2.5 m in deep, with a flat bottom formed by excavation and the heightening of the banks with removed soil to form a 1-m berm (see Figure 1(7)). Recovered materials are associated with the polychrome tradition, dating to around AD 1000. The structure in question is not yet dated. In the upper Xingú region, Heckenberger et al. (2003, p. 1711) mentioned several “wetland features, such as bridges, artificial river obstructions and ponds, raised causeways, canals, and other structures, many of which are still in use today.” Ponds located in Central Amazonia, Belterra plateau, and Lower Madeira River have well-defined circular and oval shapes. Additionally, there are also ponds resulting from the construction of other structures (i.e., mounds, raised fields, and terraces).

In such cases, aquatic animal breeding or exploitation may have occurred, because the conditions created were favorable to their survival. Examples include the crater-type depressions next to the artificial mounds, such as at the Teso dos Bichos site, on Marajó Island, dated to roughly AD 500. At this site, Schaan (2008, p. 344) postulated that these depressions at this site were used as “fishponds” to hold fish at the beginning of the dry season. Fishing in these places is still practiced today on Marajó Island. Similar crater-type depressions in southwestern Amazonia may have served an analogous purpose (Erickson and Balée 2006). Other examples of ponds and reservoirs are found in the Andes. Locally known as *q*′*ochas*, they are important for controlling water and seasonal runoff and are used as either drinking ponds or reservoirs for animals (Lane, 2014).

### Turtle corrals

Corrals are geometric structures surrounded by wood sticks, vertically arranged, forming an enclosure fence for live Giant South American River Turtles (*Podocnemis expansa*). Usually, female turtles captured in the dry season on beaches during their breeding period were stored in corrals for later consumption (Marcoy, 1875; Goeldi, 1906; Bates, 1944; Veríssimo, 1970; see Figure 1(8)). These enclosures have been described as natural or artificial lakes located in domestic backyards (Ferreira, 1903) that were used by Amerindian communities since the 15th century to conserve rainwater (Acuña 1994 [1641]). Almost all reports describing Amazonia by chroniclers and naturalists between the 16th and 17th centuries note that corrals were commonly used to store live animals (Machado, 2016: 60). In the 18th century, corrals provided the main food for the local population as well as for soldiers and the Portuguese settlers (Ferreira, 1903). Because of their great relevance in the local diet, these turtles were known by the local population as “*bois do rio,*” “river bulls” (Moll and Moll, 2004), or “Amazonian bull” (Veríssimo, 1970 [1875], Gilmore, 1997) considering the great amount of meat that they provided.

Turtles were an economically safe species providing a highly reliable source of food (Ferreira, 1903, p. 184). They could be kept in these enclosures for up to 6 months (Daniel, 2004 [1741–1757]) without the need for food and slaughtered as needed (Santos and Fiori, 2020, p. 357). In the Upper Amazon, turtles could be kept for years and they even reproduced in confinement. According to Silva-Coutinho (1868): “In the Upper Amazon an excavation is practiced in the garden which is filled with water, the turtles live there perfectly well for several years, lay eggs at the suitable time and reproduce with the greatest ease.” Although recent efforts have been undertaken to breed turtles in the Amazon, specific questions need to be addressed to better understand the return on investment of keeping turtles in the past in comparison to other species. Were they fed in corrals, and if so, with what? Contemporary studies show that *P. expansa* is herbivorous in the wild (Pritchard and Trebbau, 1984) and omnivorous in captivity (Malvasio et al., 2003). According to historical accounts, turtles kept in corrals were fed with tree branches, leaves of plants such as *aninga* (*Montrichardia linifera*), vegetables, and manioc flour (Acuña, 1994 [1641]; Silva-Coutinho, 1868; Vieira, 1970).

Despite ethnohistorical reports, from an archaeological point of view, it is difficult to say definitely that excavated structures found in Amazonia were specifically built for turtle corrals. Moraes (2006) excavated one (12 × 6 m and 1.2 m in depth) of the three ponds recorded at the Lago do Limão site (Central Amazonia), revealing stake marks on the edges of a trench opened by researchers, similar to ethnohistorical descriptions of turtle corrals (see Figure 1(2)). While radiometric dates remain to be validated, the time of construction of this particular structure is estimated to be between AD 300 and AD 1200. At the nearby, contemporaneous Hatahara site, zooarchaeological turtle remains were found. The subsequent analysis of this material showed that the remains were of individuals of the genus *Podocnemis* with an estimated length between 30 and 70 cm. This suggests a conscious prey selection guided by both the choice of taxon and the size of the individuals (Prestes-Carneiro et al., 2016).

Because Brazilian legislation prohibits capture and consumption of chelonians, except in very peculiar cases (1998), corrals are rare today. However, in the Jau river (Central Amazonia), some local villages maintain other species of chelonians (*Podocnemis erythrocephala*, *Peltocephalus dumerilianus*, and *Podocnemis unifilis*) for many months in corrals next to their residences (Pezzuti et al., 2004). It is also important to point out the existence of another type of corral, known as beach corral. This refers to a trap used on the beach edge for the capture of *P. unifilis* females in the Jaú river (Pezzuti et al., 2004; Rebêlo et al., 2005).

### Fish weirs

Here, we use “fish weir” to designate an obstruction placed in tidal waters, or wholly or partially across a river, or crossing floodplain areas to direct the passage of fish or to trap them. These structures can be made of earth, stone, or wood. Here, with a focus on storage processes, we will pay attention to structures that, in addition to allowing capture, provide some type of maintenance of animals for a period of time. The first mentions of weirs in the interior of South America are from the Baures region, Bolivia. In the extensive floodplain savannas of this region, Erickson (2000) identified kilometers of zigzag lines built of the earth (1 to 2 m wide and 20 to 50 cm tall). At the end of the savanna’s flood period, these would help to block and direct the water. These structures were interpreted as fish weirs. More recently, Blatrix et al. (2018) have shown that these earth structures are spatially associated with artificial ponds.

In other areas of the Amazon, archaeological and historical accounts recognize similar waterworks (e.g., canals), yet their relation to fishing activity remains unresolved. As with the artificial river obstructions in the Upper Xingu (Heckenberger et al., 2003) that have been in use since the occupation of the area in AD 1200, the relation to fishing is unclear. In the Venezuelan llanos, Zucchi (1984) defined channel-like structures that connect rivers as “dikes,” dated from AD 500 to AD 1400 (see Figure 1(1)). He maintains that the purpose of these constructions was to retain aquatic fauna, but no direct archaeological study has contributed to the understanding of their true function.

**Figure 3.**
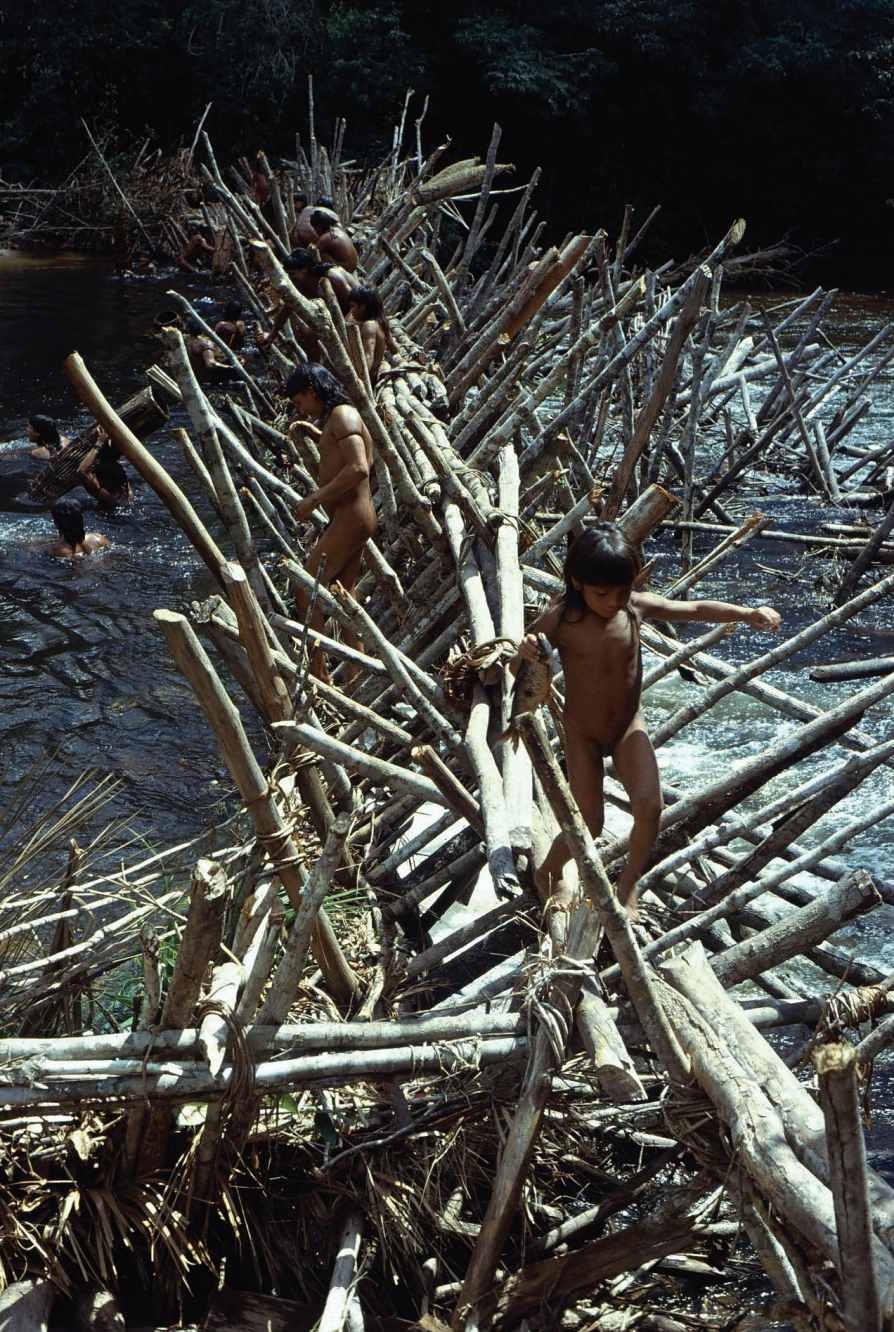
Enawene-Nawe fish weirs (Mato Grosso) (Credits: Gilton Mendes dos Santos).

In the early 20th century, Nimuendajú (2004) toured the Lower Madeira River and described river dams in Rio Pretó do Pantaleaõ and in Lake Mastro (Brazil), which that author considers to be fish dams. Contemporary inland dam use does not reflect what is seen in the archaeological record. For instance, contemporary dams made by the Tacana group of the Llanos de Mojos (Bolivia) consist of stone and earth. The Tacana plug streams and small rivers with stone and clay dikes or put a double row of reeds at the bottom of the river (Hissink and Hahn, 2000). There are many references to wooden dams built specifically for fishing that are mentioned in the ethnohistorical record among various indigenous groups in the Amazon. Well known are the fish dams made by the Enawenê-Nawê, who live in a transition region between the Cerrado and the Tropical Forest in the south of the Brazilian Amazon. Between February and April, the ceremonial practice of building the Enawenê-Nawê dam (*waity*) sees a collective mobilization and participation of the men who build it and live nearby for the duration of the project (Mendes dos Santos and Santos, 2008). These dams are built during the receding waters in rivers, when the fish leave the flooded areas and migrate to the river channels (see Figure 3).

### *Cacuris* or fish corrals

*Cacuris* are traps to catch fish and keep them alive, like in corrals, which are used throughout Amazonia (Veríssimo, 1970; Silva, 2011; Cabalzar and Candotti, 2014). Two kinds of *cacuris* have a long history of use and are still used today: one portable and the other stationary. The stationary version of the trap is built at the edges of rapids or falls, in sections of the river where the water current flows over rocky outcrops, forming waves and swirls (Cabalzar and Candotti, 2014, p. 84). Silva (2011, p. 153) writes, “It is placed at the beginning of the flood (...) capturing in great quantity the shoals (*Leporinus* spp., *Curimata* spp. and *Pimelodus* spp.) that go upstream against the current during the food migrations.” 

On the other hand, the portable *cacuri* is considered a smaller variation of the stationary *cacuri* that is used to catch small fish. The fish enter through a hole, attracted by food (mainly termites), which floats in the inner surface of the artifact (Cabalzar and Candotti, 2014, p. 76). This trap is placed near the riverbank (Cabalzar and Candotti, 2014). Silva (2011) described the use of *cacuri* to capture chelonians in places with fruit-bearing palm trees (*Mauritia flexuosa*) located near the headwaters of the Rio Negro.

### Management practices that do not imply the elaboration of physical structures

Important to point out that, while forest people likely practiced aquatic animal husbandry, it is not inherent that a group will construct an edifice to maintain species for later consumption (Veríssimo, 1970). It is known that forest people use manioc root submerged in water to attract turtles and make them “accustomed” to this procedure over several days, thereby facilitating their future handling (Barboza et al., 2013). Littoral Amazonian groups describe microhabitats on the edge of the estuary as *emburateua* that serve to shelter fish to feed and reproduce. The *emburateuas*, whether artificial or natural, are characterized by fallen debris from mangrove trees and represent important fishing spots (Barboza and Pezzuti, 2011). A further example of husbandry devoid of special structures is offered by the Katukina indigenous people in western Amazonia, who are known to keep turtles temporarily tied and maintained in puddles or in home gardens for subsequent consumption (M. Barboza, personal observation). Although turtles are kept for short periods, generally a few days, they can be fed by people in natural environments without the need for any kind of structure.

## Final Considerations

In this paper, we have presented a set of archaeological, historical, and ethnographic data that confirm aquatic environments as places of domestication scenarios. The structures built for the provisioning and captivity of animals seem to be planned using a deep knowledge of the diversity and plurality of Amazonian aquatic and terrestrial microenvironments, seasonality (water regime), land topography, quality and availability of constructive materials, and animal ecology (feeding) and behavior (trophic and reproductive migration, social interaction).

The archaeological ponds found in the Amazon region appear mainly in the seasonally flooded regions, such as the Venezuelan (Llanos Venezolanos) and Bolivian (Llanos de Mojos) savannas. The ponds that have been excavated so far in Amazonia have ranged from 12 to 38 m in diameter and from 1 to 2.5 m in depth. The water supply of these ponds could be linked to the flooding of rivers, as in the Baures systems, or precipitation such as at Loma Salvatierra and the Belterra Plateau. The function of these ponds seems to be linked to water dynamics. Perhaps they were built during the dry season—4 to 5 mo—as they can flood and retain water during the wet season. In some cases, as at Loma Salvatierra, channels ran from the highest to the lowest places, feeding the ponds with water.

Although absolute radiometric dates are not available for any of these structures, in Bolivian Amazonia, the oldest are dated to AD 300. The function of the ponds is not always clear. In Central Amazonia (Lago do Limão site), where stake marks were found, it is possible that they served as corrals for turtles. At the Belterra Plateau, interviews with contemporary residents near the archaeological sites indicate that the ponds are multifunctional. They are currently used for water supply, fish farming, and even for the introduction of other aquatic animals, such as alligators and turtles. Several features in the same site can work together as a system, and that the same structure might have more than one function. As for dam systems, there is diversity in the building material, wood, stone, or earth, but only earth and stone dams are visible in the archaeological record. Modern examples of *emburateua* and the current use of archaeological ponds in Marajó and Llanos de Mojos also demonstrate the use of traditional ecological knowledge for management strategies.

The duration of animal “captivity” and the period during which ponds can store fish is likely to show great variation. Duration seems to be quite variable depending on the structure, ranging from days to months. For example, although it is difficult to precisely affirm, we postulate that dams built of wood in rivers may only be viable for a few weeks or months. On the other hand, ponds can store fish throughout the drought period, depending on multiple factors such as the rainfall, flooding regimes, and fish taxa involved. Historical accounts suggest that turtles were stored in corrals for weeks or up to 1 yr. Thus, the structures related to the captivity of fish and turtles in the Amazon raise questions about the connection between a level of animal husbandry and the anthropogenically modified terrain, which we have termed waterscapes.

If a “classic” model of animal domestication did not occur in the Amazon, in terms of reproduction and length of captivity, clearly different cases of aquatic environmental management have existed over time in the Amazon. As suggested in the paper, some interventions and controls performed in aquatic environments depend more on daily observation and on the knowledge of the dynamics of these environments than in fact on a transformative physical intervention of the place. In these cases, it would be difficult to accurately classify the degrees of intensity. Even so, in some of the examples, as in the cases of the Baures region or the Marajó island, waterscape domestication allowed populations to permanently transform environments that would be seasonally or completely flooded or completely dry. Waterscape domestication allowed these populations to live out of water in flooded environments and to continue to manage water and aquatic fauna in periods that would naturally be absent. Therefore, perhaps what we are able to observe more accurately is the persistence of some changes over time.

The domestication that could appear to be “incomplete” from a western point of view seems to have been intentional, through a more in-depth, integrated, and engaged ontology. An example of this is indigenous perception of intangible beings—animals, plants, stones, spirits, etc. (Viveiros de Castro, 1998). Thus, the action of managing these environments also involves negotiating, collaboration, respect, and experimentation with multiple beings and the spaces they inhabit across time (Mendes dos Santos and Santos, 2008; Barboza et al., 2021). In this text, we have argued for the dialogue between waterscapes and associated parts (humans, tangible, and intangible beings). We further argued for the role of waterscapes in the archaeological and historical past on the ongoing building of Amazonian landscapes.

Evidence of water-managed structures are scattered throughout the archaeological and historical record; therefore, it will be important for the relationship between people and the Amazon that previous water technologies are revisited and systematically studied. To recognize the living memory of the people who built the forest, researchers need to understand the dimension of the waterscape. This is possible knowing the beings that inhabit it, the dynamics of their interaction, and the social complexity of human interactions with the environment and other beings. Most important is working with local people, who embody the deep socio-ecological knowledge of Amazonia. These collaborative efforts will help identify which traditional methods can persist in the future and can be useful to deal with current problems resulting from unsustainable practices (Krenak, 2019).

## About the Authors

**Gabriela Prestes-Carneiro** (PhD) is an assistant professor in the Anthropology and Archaeology Department at the Federal University of Western Pará State (UFOPA), in Santarém, Brazil. She is an Amazonian zooarchaeologist who has worked on archaeological shell mounds, anthropogenic dark earths, and monumental mounds in different regions of the Brazilian and Bolivian Amazon. Fish remains have been her primary specialty as she explores histories of the human use of the aquatic environments, subsistence changes, and their relationships to the paleoenvironment.

**Roberta Sá Leitão Barboza** (PhD) is a biologist specialized in Aquatic and Fish Ecology at the Federal University of Pará (UFPA) and has been working in the Amazon with artisanal fishermen since 2004 and teaching as a university professor for over 10 yr (UFPA). Roberta has developed studies on the uses, fishing, and community management of turtles in Amazon. Since 2014, she coordinates the research group Coastal Socio-Environmental Studies and the Laboratory of Teaching, Research and Fisheries Extension for Amazonian Communities. Currently, she develops scientific studies (Fishermen’s Social Organization, Local Ecological Knowledge, and Participative Management of Natural Resources) and scientific dissemination products for traditional communities in the Amazon.

**Myrian Sá Leitão Barboza** (PhD) is a biologist specialized in Cultural Anthropology and Latin-American Studies (University of Florida) and has been working in Amazonia since 2004. She has been an assistant professor at the Federal University of Western Pará State (UFOPA), in Santarém, Brazil, for over 10 yr, where she currently coordinates the Archaeology course. She has experience with ecological, ethnoecological, and ethnographic fieldwork with different groups (indigenous, Afro-Brazilian, riverine, women, and social movements) in Brazilian Amazonia and the Northeast. Currently, Myrian is engaged with local communities on studies about past and present diets, community use and management of animal and plant resources, and gendered use of territory.

**Claide de Paula Moraes** (PhD) is a professor of Archeology at the Federal University of Western Pará. He has experience in Amazonian Archeology, active mainly in the following research themes: peopling of the Americas, the year AD 1000 in the Amazon, the study of ceramic sites, study of lithic technology, indigenous people and archaeology, and teaching of archeology.

**Philippe Béarez** (PhD) is an archaeozoologist specialized in the study of interactions between humans and aquatic environments over time. Fish are his favorite animal group, which he studies in several ways: archaeoichthyology, anatomy, taxonomy, biogeography, and conservation. He is also interested in historical ecology and human impacts on aquatic ecosystems. He carries out archaeological work in South America (east coast of the Pacific, Amazonia), in the Indian Ocean (Arabian Sea coast), in Oceania (Melanesia and Polynesia), and in France.
